# *Salmonella *Typhimurium invasion of HEp-2 epithelial cells *in vitro *is increased by *N*-acylhomoserine lactone quorum sensing signals

**DOI:** 10.1186/1751-0147-53-44

**Published:** 2011-06-28

**Authors:** Live L Nesse, Kristin Berg, Lene K Vestby, Ingrid Olsaker, Berit Djønne

**Affiliations:** 1 Norwegian Veterinary Institute, P.O.Box 750 Sentrum, N-0106 Oslo, Norway; 2 Norwegian School of Veterinary Science, P.O.Box 8146 Dep, N-0033 Oslo, Norway

## Abstract

**Background:**

In Gram-negative bacteria, the most commonly studied quorum sensing signals are the *N*-acylhomoserine lactones (AHLs). In *Salmonella*, AHLs are recognized by SdiA, which is believed to be a sensor of AHLs produced by other bacteria, since *Salmonella *does not produce AHLs itself. It has been speculated that AHLs produced by the gastrointestinal flora may influence the regulation of virulence traits in *Salmonella*. The aim of the present work was to study the effect of AHLs on epithelial cell invasion by *Salmonella in vitro*.

**Methods:**

Invasion by *Salmonella enterica *subspecies *enterica *serovar Typhimurium (*S*. Typhimurium) strain and its isogenc sdiA mutant was studied using a conventional gentamycin invasion assay with HEp-2 cells at 37°C. Gene expression was studied using a semi-quantitative PCR.

**Results:**

The *S*. Typhimurium strain, but not its isogenic *sdiA *mutant, displayed increased *in vitro *invasion after addition of both *N*-hexanoyl-DL-homoserine lactone (C6-AHL) and *N*-octanoyl-DL-homoserine lactone (C8-AHL). Increased expression of two of the genes in the SdiA regulon (*rck *and *srgE*) was observed in the wild type strain, but not in the *sdiA *mutant.

**Conclusions:**

The results from the present study show that *S*. Typhimurium can respond to two different AHL quorum sensing signals (C6-AHL and C8-AHL) with increased cell invasion at 37°C *in vitro*, and that this response most likely is *sdiA *mediated. These results indicate that if AHLs are present in the intestinal environment, they may increase the invasiveness of *Salmonella*.

## Introduction

Bacteria can communicate through quorum sensing signals, and they use quorum sensing to regulate a number of physiological activities, e.g. symbiosis, virulence, competence, conjugation, antibiotic production, motility, sporulation, and biofilm formation (for review, see [1,2]). In Gram-negative bacteria, the most commonly studied quorum sensing signals are the *N*-acylhomoserine lactones (AHLs). A variety of AHL molecules have been discovered which differ primarily in acyl chain length and the nature of the substituents at the C-3 position. AHLs are synthesized by proteins encoded by *luxI *gene homologues.

*Salmonella enterica *is a facultative intracellular pathogen that can cause diseases ranging from mild gastroenteritis to systemic infections. The AHL sensor of *Salmonella *is *sdiA *[3]. It is believed that *Salmonella *acquired the *sdiA *gene through lateral transfer of a pseudomonad homologue to an early ancestor [4]. However, searches in the existing databases have failed to identify any *luxI *homologue in available sequences [3], indicating that *Salmonella *do not synthesize AHLs. This is supported by studies showing that *Salmonella *did not activate any AHL reporter systems tested under the conditions employed [3].

Consequently, *Salmonella *appear to be able to recognize AHL signals, but not to produce them. SdiA is therefore believed to be a sensor of AHLs produced by other bacterial species [3], possibly in the mammalian gastrointestinal tract [5]. An interesting question is whether AHLs produced by the gastrointestinal flora may influence the regulation of virulence traits in *Salmonella *like cell invasion. In the present work, the effect of different AHLs on invasion of HEp-2 epithelial cells by *Salmonella enterica *subspecies *enterica *serovar Typhimurium (*S*. Typhimurium) was studied *in vitro*.

## Materials and methods

### Bacterial strains

The bacteria used in this study were the wildtype *S*. Typhimurium ATCC 14028 and its isogenic *sdiA *mutant (ΔSTM1950: Kan-PT7) which was kindly provided by Professor McClelland at Vaccine Research Institute of San Diego, USA. As a control bacterium, known to be non-invasive, *Escherichia coli *ATCC 25922 was included. Cultures were routinely grown in 5 mL Luria-Bertani (LB) (Merck KGaA, Darmstadt, Germany) broth without agitation for approximately 18-20 hours at 37°C before used in experiments.

### Cell lines, culture conditions and buffers

The cell line HEp-2 ATCC CCL-23 (LGC Standards, Middlesex, UK) was used in the invasion experiments. The cells were grown in Minimum Essential Medium (MEM; Lonza, Basel, Switzerland) supplemented 2 mM L-glutamine (Lonza) and 10% fetal calf serum (Merck) at 37°C under standard tissue culture conditions, without CO_2 _in 25 cm^2 ^flasks (Corning B.V. Lifesciences, Amsterdam, Netherlands) and confluent flasks were split twice a week by trypsin-EDTA (Lonza) treatment and diluted 1:8 in fresh media. When used for bacterial invasion studies, the cells were diluted 1:2, seeded in 24 well plates (Corning) and incubated over night at 37°C. The following day, cells were counted using a Bürker chamber after staining with Trypan Blue Stain 0.4% (Lonza) to quantify the mean number of cells per well.

### Cell invasion studies

Over night cultures were diluted 1:100 in 5 mL LB and subcultured for 3 hours in 37°C. Then the cultures were ten fold diluted twice, first in MEM and subsequently in MEM supplemented with either *N*-hexanoyl-DL-homoserine lactone (C6-AHL) (Sigma-Aldrich, St. Louis, MO, USA) or *N*-octanoyl-DL-homoserine lactone (C8-AHL) (Sigma-Aldrich) to a final concentration of 1 μM/mL (hereafter called MEMs). Distilled water (dH_2_O) was used instead of AHL for the controls. To measure bacterial invasion, a method based on the one described by Lissner *et al*. was used [6]. Briefly: 1 mL bacteria/MEMs was added to HEp-2 cells grown over night in 24 well plates, to a multiplicity of infection (MOI) of approximately 100 bacteria pr cell. Plates were incubated for 90 min at 37°C and MEMs was removed. The cells were washed three times with 1 mL PBS de Boer (Na_2_HPO_4 _× 2H_2_O 1.34 g/L, NaH_2_PO_4 _× H_2_O 0.34 g/L, NaCl 8.5 g/L, pH: 7.2 ± 0.1). Then 1 mL MEM supplemented with 250 μg/mL gentamycin (Lonza) (hereafter called MEMg) was added, followed by incubation at 37°C for two hours. MEMg was removed; cells were washed three times with PBS de Boer and lysed with ice-cold 1% Triton-X (Sigma-Aldrich) in PBS de Boer for 10 min. The cells were dislodged using a sterile cell scraper (BD Falcon, Bedford, MA, USA), pipetted to disperse bacterial aggregates, ten fold serial diluted and plated out on blood agar to determine the number of cfu.

In each experiment, every combination of supplement and bacteria were tested in triplets, and three independent experiments were performed for each strain. To eliminate the day to day variations, the effect of AHL addition in each experiment was calculated as fold change, i.e.: (the mean number of intracellular cfu after addition of AHL)/(the mean number of intracellular cfu without addition of AHL), and thereafter log_10 _transformed to allow calculation of confidence intervals. Results are given as means of three independent experiments. An increase in cell invasion was considered statistically significant (p <0.05) if the value "0" was not included in the 95% confidence interval.

### Semi-quantitative PCR

The bacteria were prepared and incubated with 1 μM AHL or dH_2_O as described under cell invasion studies with the exceptions that HEp-2 cells were not present, and the incubation was performed in 100 mL LB. After incubation, each bacterial suspension was divided into 25 mL aliquots and transferred to four 50 mL test tubes (Greiner Bio-One, Frickenhausen, Germany). To each tube, 5 mL ice cold 5% acidic phenol (Sigma Aldrich)/95% ethanol (Kemetyl Norge, Vestby, Norway) were added and the mixture was kept on ice for 20 min to stabilize the mRNA [7]. The mixture was then centrifuged at 4°C, 2330 × G for 20 min, and most of the supernatant discarded. Each pellet was resuspended in the remaining supernatant. The suspensions were pooled two and two, transferred to two Eppendorf tubes (BRAND GMBH, Wertheim, Germany) and centrifuged at 14 000 × G for 1 min at room temperature. The supernatants were discarded and the pellets frozen at -70°C until the next step in the procedure. Total RNA was isolated from the pellets using a SV Total RNA Isolation kit (Promega Corporation, Madison, WI, USA) according to the manufacturer's instructions. cDNA was synthesized immediately after RNA isolation, using Invitrogen's SuperScript^® ^II Reverse Transcriptase (Invitrogen, Ltd, Paisley, UK) according to manufacturer's instructions. PCR was performed in 25 μL reaction volumes using1 μL cDNA, 0.5 μL of each primer, 0.5 μL of each dNTP and 0.2 μL Taq polymerase (Qiagen GmbH, Hilden, Germany), and thermocycled by 5 min initial denaturation at 95°C, thereafter 26, 28 and 31 cycles of 95°C 40s, 60°C 30s and 72°C 40s, followed by 7 min final extension at 72°C. The primers used are listed in Table 1. The PCR fragments were separated by gel electrophoresis and examined both visually and using BioNumerics^® ^software (Applied MathsBVBA, Belgium). The best measurements of quantitative differences were obeserved at 28 cycles.

**Table 1:** Primers used for RT-PCR

Gene	Acc. no/locus tag	Primers 5' - 3' F: forward R: reverse
***rck***	CP001362.1 /STM14_5534	F: GTTGTATCCCGGCATGCTGAT R: ATATGCCCAGAGCCGGATAGAG
***srgE ***	AE006468.1/STM1554	F: GTAATGTCAATTGCGGCATGG R: CGGAGCAGTTGGTCAAGGATT

## Results and Discussion

**Figure 1:**
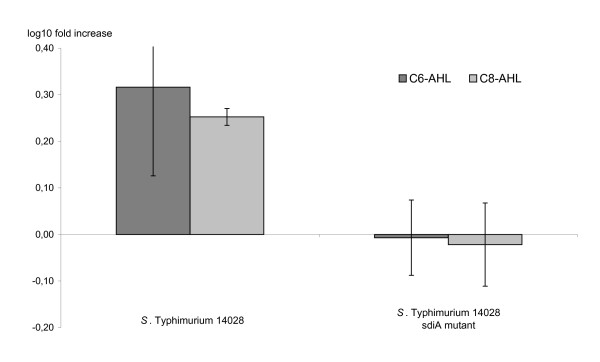
**The effect of *N-*hexanoyl-DL-homoserine lactone (C6-AHL) and *N-*octanoyl-DL-homoserine lactone (C8-AHL) on cell invasion by *Salmonella *expressed as mean fold increase (log_10 _transformed)**. Bars show 95% confidence interval. When the value 0 is not included in the confidence interval, the increase is considered statistically significant, i.e. p <0.05.

Both the *S*. Typhimurium wildtype strain ATCC 14028 and its isogenic *sdiA *mutant displayed invasion of the epithelial cell line HEp-2 at 37°C without addition of AHLs (0.5 - 2.5 * 10^5 ^cfu per well, depending on the day to day variation). However, when 1 μM of C8-AHL or C6-AHL was added, the *S*. Typhimurium wildtype strain displayed statistically significant higher cell invasion (approximately two fold) with both AHLs, as compared to the invasion without AHL (Figure 1). No such differences were observed when testing the isogenic *sdiA *mutant strain, indicating that the increased cell invasion responses were *sdiA *dependent.

**Figure 2:**
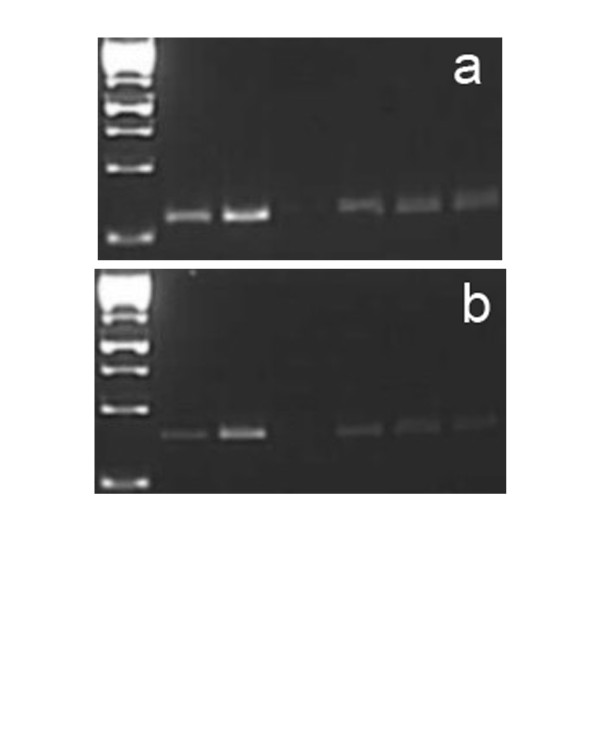
**Results from semi quantitative PCR run 28 cycles**. a) *rck *b) *srgE*. In both pictures: lane 1: 1 kb ladder, lane 2: 14028 wild type with AHL-C6, lane 3: 14028 wild type with AHL-C8, lane 4: 14028 wild type with dH_2_O, lane 5: 14028 *sdiA *mutant with AHL-C6, lane 6: 14028 *sdiA *mutant with AHL-C8, lane 7: lane 5: 114028 *sdiA *mutant with dH_2_O.

To date, SdiA is known to activate two loci, containing a total of seven genes [5,8]. One locus, the *rck *operon, is located on the virulence plasmid and contains six genes. The second locus is located on the chromosome and encodes a single gene, named *srgE*. Several different AHLs, including C6-AHL at the concentration of 1 μM, have earlier been shown to activate promotors of the genes *rck *and *srgE *in an *sdiA*-dependent manner in *Salmonella *[5,8]. To see if these genes were activated in our experiment, we studied the expression of the genes under similar experimental conditions as the cell invasion experiments using a semi-quantitative PCR (Figure 2). For both genes, we observed a low level of *sdiA*- and AHL-independent expression, as earlier reported for *rck *[5,8]. In addition, the wildtype *S*. Typhimurium strain displayed an increased expression of both *rck *and *srgE *when 1 μM of C8-AHL or C6-AHL was added, whereas its isogenic *sdiA *mutant did not. The results indicate that SdiA acted as an AHL induced transcriptional regulator under our experimental conditions.

Consequently, the results from the present study show that a *S*. Typhimurium wildtype strain did respond to both C8-AHL and C6-AHL with increased epithelial cell invasion at 37°C *in vitro*, most probably through activation of SdiA.

The exact mechanisms behind the increased cell invasion that we observed are not known. However, several genes regulated by SdiA are believed to be involved in bacteria-host interactions. Three genes in the SdiA regulated *rck *operon play a role in adhesion to host tissues [8]. It has earlier been shown that Rck promotes adherence to epithelial cells and the extracellular matrix proteins fibronectin and laminin [9], and Rck has recently also been reported to mediate a zipper-like internalization of *S*. Enteritidis into cells *in vitro *[10]. Two other genes in the *rck *operon, *pefI *and *srgA*, appear to affect the expression and function of the *pef *operon which encodes socalled plasmid-encoded fimbriae [9,11,12]. The function of the rest of the genes in this operon is unknown. Very little is also known about *srgE*, but recently a computerized analysis suggested that SrgE may be a secreted substrate of a type III secretion system [13].

It has earlier been suggested that the mammalian gastrointestinal tract may be the location where SdiA detects and responds to AHLs [5]. Although compounds that can activate AHL biosensors have been detected in the bovine rumen [14] and the avian craw (Flodgaard, Nesse, Bergsjø & Kaldhusdahl, unpublished results), little is yet known about the AHL producing potential of the intestinal flora of different hosts under varying conditions. Using a RIVETmethod (Recombination-based In Vivo Expression Technology) to record SdiA activity *in vivo*, Smith *et al*. observed SdiA activation during the transit of the *S*. Typhimurum RIVET strain through turtles colonized by the AHL-producing species *Aeromonas hydrophila *[15]. On the other hand, SdiA activation was not observed during the transit through the gastrointestinal tract of a guinea pig, a rabbit, a cow, five mice, six pigs, or 12 chickens [15]. Interestingly, SdiA was activated in mice that were infected with the AHL-producing pathogen *Yersinia enterocolitica *[16]. These results indicate that *S*. Typhimurium can respond to AHLs present in the intestinal environment of these animals through the activation of SdiA.

## Conclusions

The results from the present study show that *S*. Typhimurium can respond to two different AHL quorum sensing signals (C6-AHL and C8-AHL) with increased cell invasion at 37°C *in vitro*, and that this response most likely is *sdiA *mediated. This indicates that if AHLs are present in the intestinal environment, they may increase the invasiveness of *S*. Typhimurium into epithelial cells. However, any possible effects on virulence are yet to be elucidated.

## Competing interests

The authors declare that they have no competing interests.

## Authors' contributions

LLN was responsible for the study design, organisation of the work, analyses of the data and the preparation of the manuscript. KB carried out the cell invasion studies and PCR studies. LKV participated in the study design and the data analyses. IO was responsible for primer design and contributed to the PCR analyses. BD contributed to study design and participated in the data analyses. All authors have contributed to the writing of the manuscript, and read and approved the final manuscript.
